# Influence of the filler distribution on PDMS-graphene based nanocomposites selected properties

**DOI:** 10.1038/s41598-022-23735-3

**Published:** 2022-11-09

**Authors:** Anna Łapińska, Natalia Grochowska, Jerzy Antonowicz, Przemysław Michalski, Kamil Dydek, Anna Dużyńska, Agata Daniszewska, Milena Ojrzyńska, Klaudia Zeranska, Mariusz Zdrojek

**Affiliations:** 1 Faculty of Physics, Warsaw University of Technology, Koszykowa 75, 00-662 Warsaw, Poland; 2 Faculty of Materials Science and Engineering, Warsaw University of Technology, Wołoska 141, 02-507 Warsaw, Poland

**Keywords:** Composites, Structural properties, Synthesis and processing

## Abstract

Insufficient homogeneity is one of the pressing problems in nanocomposites’ production as it largely impairs the properties of materials with relatively high filler concentration. Within this work, it is demonstrated how selected mixing techniques (magnetic mixer stirring, calendaring and microfluidization) affect filler distribution in poly(dimethylsiloxane)-graphene based nanocomposites and, consequently, their properties. The differences were assessed via imaging and thermal techniques, i.a. Raman spectroscopy, differential scanning calorimetry and thermogravimetry. As microfluidization proved to provide the best homogenization, it was used to prepare nanocomposites of different filler concentration, whose structural and thermal properties were investigated. The results show that the concentration of graphene significantly affects polymer chain mobility, grain sizes, defect density and cross-linking level. Both factors considered in this work considerably influence thermal stability and other features which are crucial for application in electronics, EMI shielding, thermal interface materials etc.

## Introduction

Nowadays, polymer nanocomposites are an integral part of human life and can be found in food packaging^1^, medicine^2^, sensors and energy harvestings devices^3^, wearable electronic devices^4^, aerospace^5^, EMI shielding^6,7^ microwave signal processing devices^8^ or thermal interface materials^9^.

However, as they consist of at least two components of different dimensionalities, it is critical to achieve sufficient filler dispersion in a polymer matrix^10,11^. Without reaching the appropriate level of filler dispersion, such material in fact has no application potential and could even be dangerous if applied^12^.

Nowadays, graphene has arisen as one of the most chosen fillers for nanocomposite design. Nevertheless, inter-layer van der Waals forces as well as weak interaction with polymer matrix make achieving homogeneity in the case of graphene a difficult task. Graphene sheets tend to agglomerate and re-stack, thus diminishing specific surface area and making it less likely to create a consistent, conductive network or path within a nanocomposite. Therefore, to make the most of the filler a proper processing method must be chosen^13^.

Many different routes for preparing graphene-polymer nanocomposites have been developed. Trivedi and Rachchh^14^ divided them into several groups such as dip-coating, casting, spray deposition, vacuum infiltration (which should include graphene foam infiltration), solution mixing, melt mixing and in-situ polymerization. Melt mixing seems to be the simplest method, it is also easily scalable and requires no solvents, which makes it economical and environmentally friendly. It faces, however, a problem of increased viscosity of the mixture, which can be attributed to large filler-matrix surface area and consequent interaction at the interface. Using a solvent facilitates processing of the mixture and can allow to break up agglomerates more effectively.

According to literature, one of the most frequently used methods to mix graphene with the polymer matrix is sonication of a pre-mixed suspension followed by solvent evaporation^15–17^. However, harsh and toxic solvents such as cyclohexane and N-methylpirrolidone are mainly used. To decrease the harmful influence of such compounds on human health during the nanocomposite manufacture, lower the cost of the process and simultaneously guarantee high filler dispersion level, a modified solvent assistant method of elastomer/graphene nanocomposite fabrication had been proposed within this work.

To do so, firstly, three different routes for processing GNP-polymer nanocomposites have been chosen and compared: simple magnetic mixer stirring (for reference), calendaring with no additional solvent and microfluidization, using a safe and cheap solvent. Calendaring falls into melt mixing category, it is commonly used in thermoplastics processing. During the process the material is passed through a narrow gap between rotating rollers and thus mixed. Microfluidization (otherwise high-pressure homogenization), on the other hand, requires solvent addition. It consists of forcing the material through a microchannel and this way subjecting it to high shear stress. The technique had been previously utilized for exfoliating graphite and breaking up agglomerates of carbon nanotubes in polymer nanocomposites^18^, hence it may be promising also in terms of graphene nanocomposite homogenization.

According to the selected manufacture methods, poly(dimethylsiloxane) (PDMS) was chosen as a matrix. It is a thermosetting resin, that could be easily subjected to calendaring with no additional solvent and at the same time could be easily dissolved in isopropyl alcohol. Additionally, its high electrical resistivity, dielectric strength, thermal and chemical resistance as well as ability of vibration damping contributes to its popularity in electronic industry, which made PDMS a suitable benchmark for analysis^17^. To sustain the elasticity of the material and its good adherence to other components the filler content was kept down to 10%wt.

Within this work, a deep insight into the influence of nanofiller distribution in polymer matrix on the properties of nanocomposites is presented. The materials’ structures were studied using polarizing-interference microscopy, scanning electron microscopy and Raman spectroscopy mapping. Raman mapping as an imaging technique directly revealed the level of sample homogenization. Additionally, differential scanning calorimetry discovered dramatic change in thermograms of samples fabricated with different techniques.

Hence, these results were motivation to take an extensive look at the influence of filler concentration on samples fabricated via microfluidization. For this purpose, samples with 1–10%wt graphene concentration were manufactured and subsequently studied using differential scanning calorimetry, thermogravimetry and X-ray diffractometry. Increasing graphene concentration in nanocomposites caused a fall in the degree of crystallinity as a result of carbon presence. The TGA and DSC analysis showed the enhancement of thermal stability and significant changes in chain mobility resulting from interaction with filler particles.

## Results and discussion

**Figure 1:**
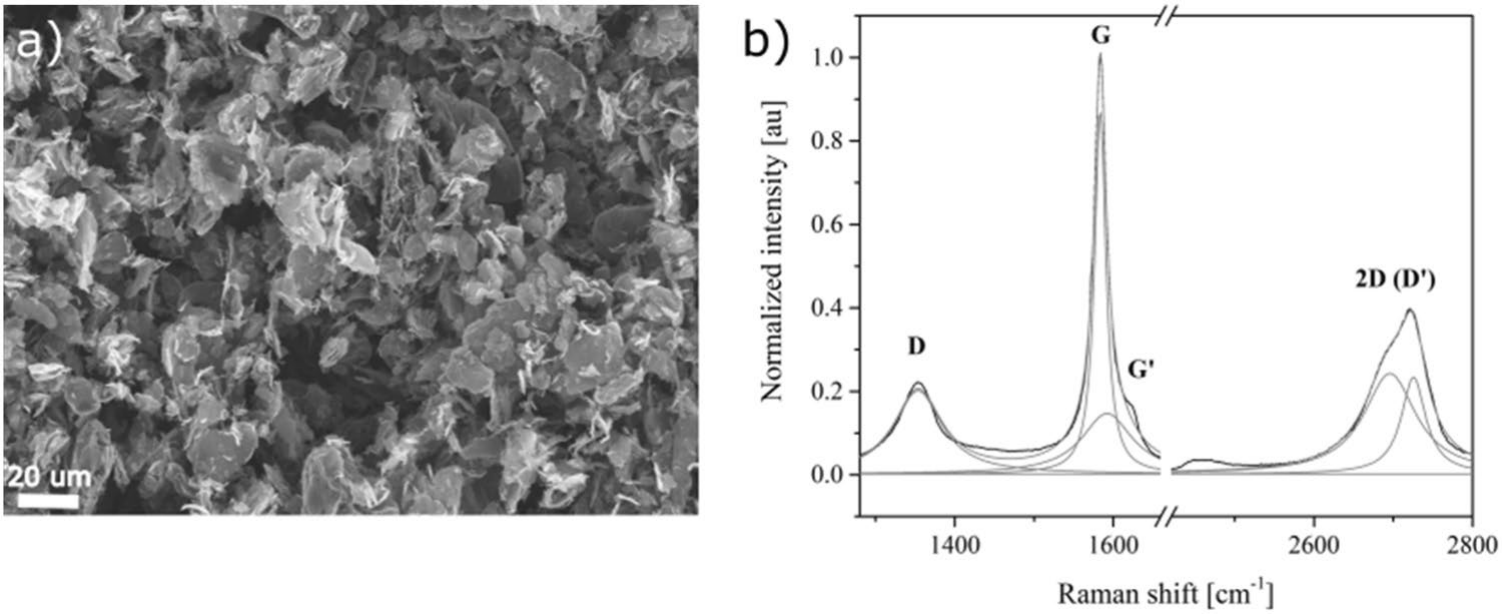
SEM photograph (**a**) and Raman spectrum with band assignment (**b**) of agglomerates of xGnP H-5 (XG Sciences, Inc., USA) graphene nanoplatelets, which were later used as a filler.

At the beginning the graphene nanoplatelets, which were chosen as a filler, were examined. Their structure was investigated through SEM (Raith eLine Plus) and Raman spectroscopy (Renishaw inVia). SEM observations (Fig. 1a) reveal that the platelets form agglomerates of diverse sizes and irregular shapes. The particles’ surface is developed, and the edges of carbon layers are often rolled up. High density of structural defects is confirmed by intensive and wide G′ peak of the Raman spectrum (Fig. 1b). These results show the complex nature of the filler which is neither graphite nor graphene in the strict sense of the term. Nevertheless, in the work the expression “graphene nanoplatelets” will be used to highlight that the effects observed are more typical of nanomaterials and the results are applicable especially to nanocomposites produced from commercial “graphene” fillers.

**Figure 2:**
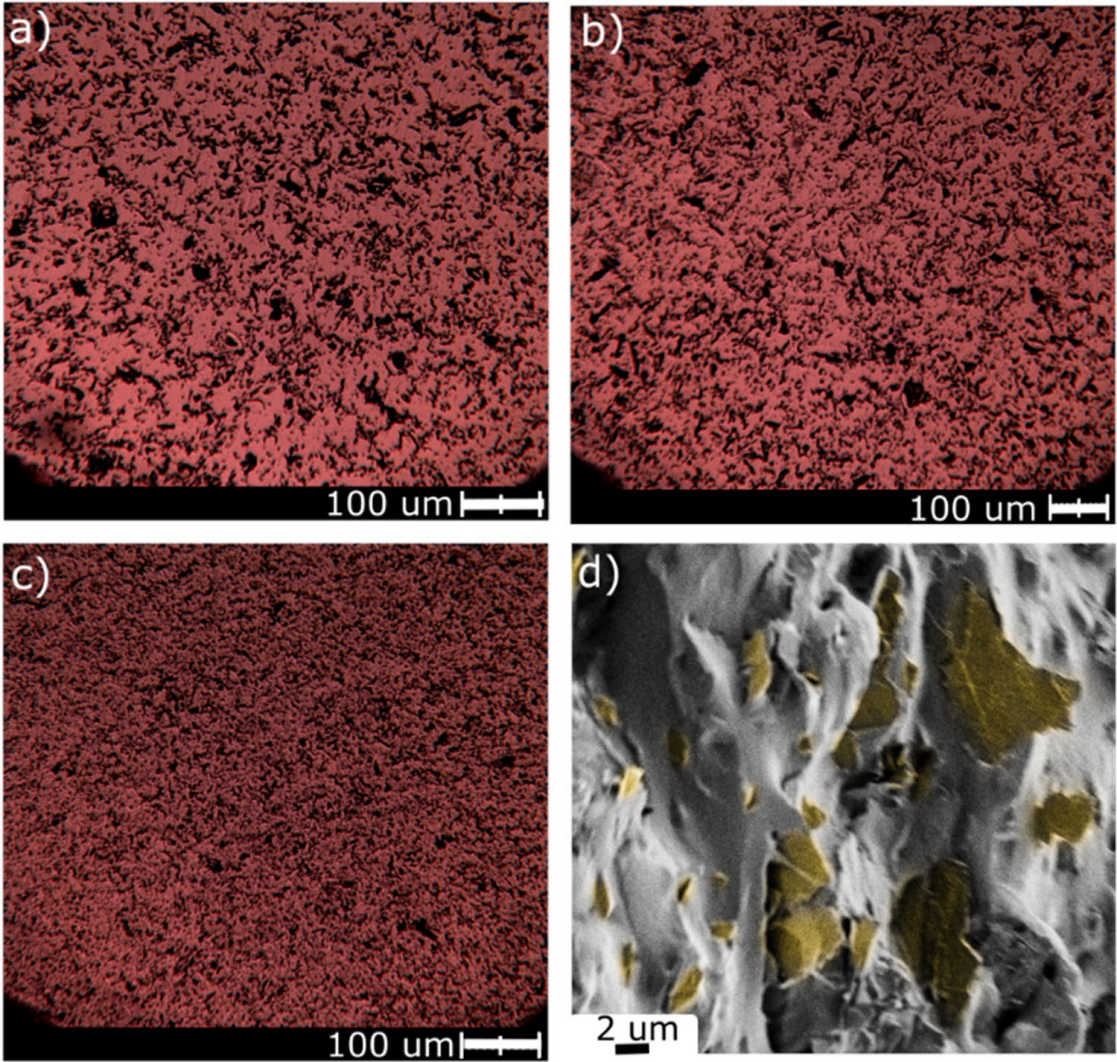
Polarizing-interference microscopy photographs of samples containing 10%wt graphene: (**a**) sample PDMS M (magnetic mixer stirring), (**b**) sample PDMS K (magnetic mixer stirring plus calendaring), (**c**) sample PDMS HPH (microfluidization), (**d**) a representative SEM scan of the PDMS/graphene nanocomposite sample (here: PDMS HPH). Yellow color refers to graphene nanoplatelets.

The structure and topography of the nanocomposites were investigated based on polarizing-interference microscopy (BIOLAR PI) and SEM. The results are shown sequentially in Fig. 2.

The sample PDMS M prepared by 5-h magnetic mixer stirring (Fig. 2a) appears less homogenized and rather big agglomerates are present. Additional calendaring (Fig. 2b) slightly improves the nanofiller dispersion in the polymer matrix, but the changes are not significant. Clear enhancement is noticed in a sample fabricated by microfluidization (Fig. 2c), where a reasonable level of dispersion and agglomerate disintegration is observed. On the other hand, SEM scans (Fig. 2d) indicate that polymer matrix sticks very well to the nanofiller flakes. These factors may contribute to difficulties in creating the conductive percolation paths and in the aftermath strongly impact the final properties of the nanocomposite.

**Figure 3:**
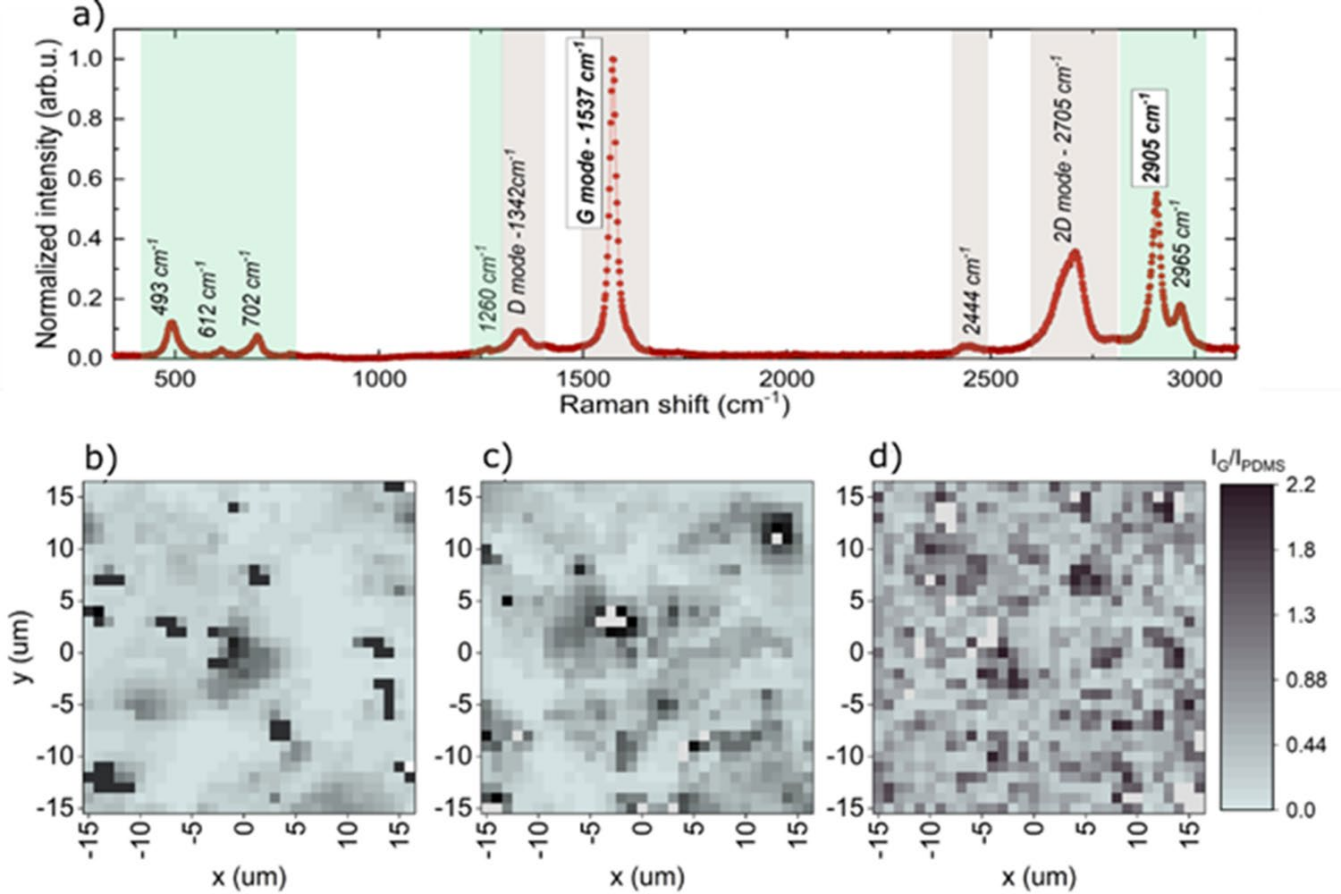
(**a**) A typical Raman spectrum of graphene/PDMS nanocomposite induced by laser wavelength λ = 532 nm at room temperature. Light green areas indicate the most prominent PDMS-based Raman modes, light grey areas indicate graphene-based Raman modes, (**b**) Raman maps of normalized intensity ratio of graphene G-band (1573 cm^−1^) to the polymer PDMS band (2905 cm^−1^) of sample PDMS M, (**c**) sample PDMS K, (**d**) sample PDMS HPH.

Raman mapping was performed to detect the distribution of graphene flakes in the PDMS matrix in higher magnification than for polarizing-interference microscopy. Figure 3 shows a typical Raman spectrum of a graphene/PDMS nanocomposite. PDMS modes were observed at the following wavenumbers (cm^−1^): 493—Si–O–Si stretching vibration, 702—Si–C stretching vibration, 1263—CH_3_ asymmetric bending vibration, 1411—CH_3_ asymmetric bending vibration, 2905—CH_3_ stretching vibration, 2965—CH_3_ stretching vibration^18,19^. Graphene modes were observed at the following wavenumbers (cm^−1^): 1342—assigned as D mode, breathing vibration A_1g_ of aromatic ring, 1537—G mode, stretching vibration E_2g_ of sp^2^ carbon pairs, 2705—2D mode, overtone of D mode^20^. An analysis of Raman mapping in terms of intensity ratio of graphene G-band and PDMS 2905 cm^−1^ band is shown in Fig. 3b–d. The selected bands were the most intensive Raman modes for graphene and PDMS, respectively. Dark black regions refer to places with predominance of graphene and light blue-grey regions represent the polymer matrix. It can be clearly seen that after the microfluidization process (sample PDMS HPH) graphene sheets exhibited a homogeneous distribution in PDMS without symptoms of severe agglomeration. Samples prepared without microfluidization (especially sample PDMS M) are heterogeneous with predomination of relatively large carbon material agglomerates, which confirmed results observed in Fig. 2 for polarizing-interference microscopy. This supports the thesis that the microfluidization process allowed to obtain better distribution of graphene filler in PDMS matrix, compared to plain mixing in magnetic stirrer or calendaring.

The melt mixing processes might have failed since the unmodified filler’s compatibility with PDMS is limited. According to Dennis et al.^21^ agglomerates of layered organic clays which are incompatible with the matrix, can be disintegrated during melt processing only to a limited extent—regardless of the applied shear stress. In the case of graphene and PDMS, even though PDMS offers good wettability, inter-layer van der Waals forces are not disrupted, and platelet delamination is not observed.

**Figure 4:**
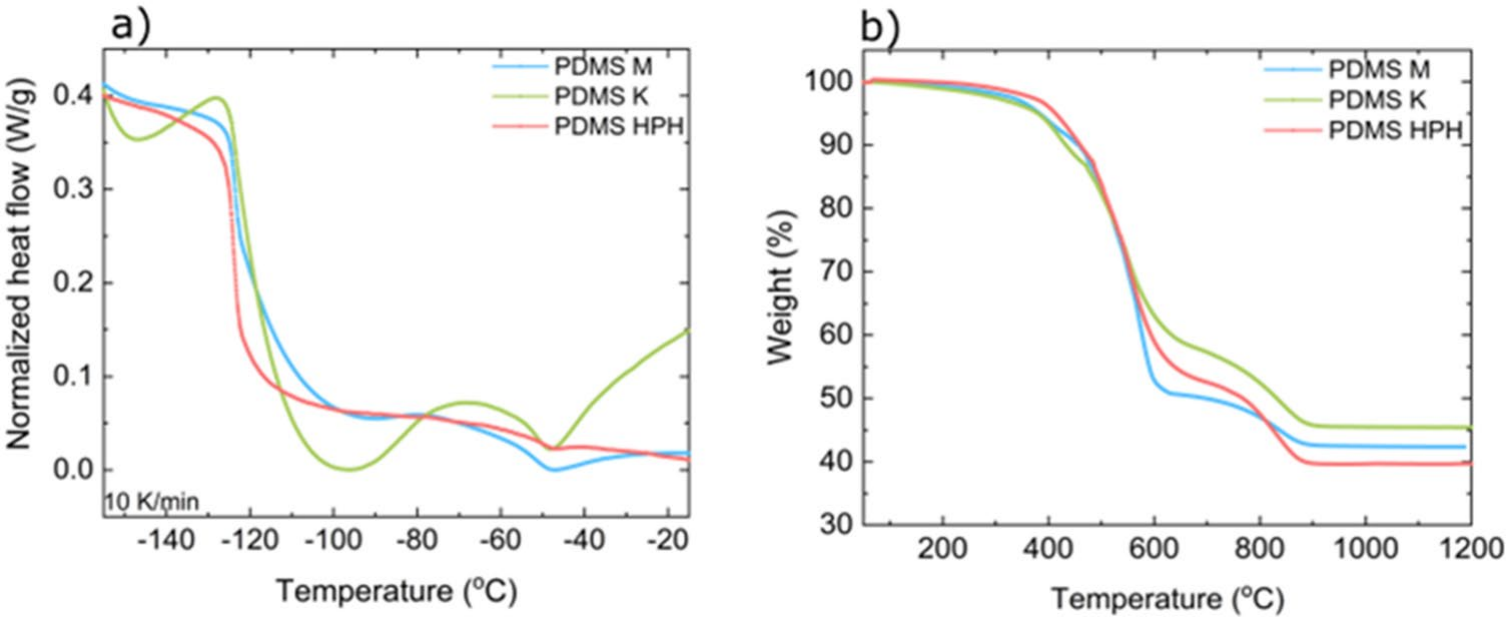
(**a**) DSC and (**b**) TGA thermograms of PDMS/10%wt graphene nanocomposite samples produced using different techniques: magnetic mixer stirring, calendaring and microfluidization.

To investigate thermal properties variation originating in different filler distribution, differential scanning calorimetry and thermogravimetry were performed. The DSC analysis concerns the analysis of the first heating cycle. Such an approach is justified as there is no need to remove the thermal history for PDMS—room temperature is high enough to keep it in the melt state^22^. Figure 4a shows DSC thermograms for 10%wt GNP samples obtained by the three methods. Additionally, Table 1 summarizes typical thermal properties values calculated on the basis of the graphs shown.

**Table 1:** Summary of typical thermal properties values coming from DSC and TGA measurements for samples produced via different routes.

k = 10 °C/min	T_g_ [°C]	ΔC_p_ [J/(g °C]	T_m_ [°C]	H_m_ [J/g]	C_p_ [J/(g °C)]	T_max1_ [°C]	T_max2_ [°C]	T_max3_ [°C]	T_max4_ [°C]
PDMS M	− 125.18	0.3562	− 48.88	0.5492	1.4600	393.01	506.61	576.75	839.66
PDMS K	− 124.87	0.3638	− 47.27	0.4632	1.4129	419.86	494.56	549.67	836.79
PDMS HPH	− 124.0	0.3555	− 47.02	0.1156	1.4600	450.85	505.16	553.75	836.55

The speed of heat remained constant: 10 °C/min. C_p_ was measured in quasi-isothermal mode during MT-DSC scan. *T*_*g*_ glass transition, *ΔC*_*p*_ heat capacity change, *T*_*m*_ melting temperature, *H*_*m*_ melting enthalpy, *C*_*p*_ heat capacity, *T*_*max1-4*_ decomposition temperatures.

Having regard to those results (Fig. 4 and Table 1), firstly, there is a dramatic change in DSC thermograms’ shape depending on filler distribution. Even though all the samples consist of the same, constant graphene filler loading of 10%wt, the melting phenomena differ in respective samples. This is also confirmed by melting temperature, which is higher for PDMS HPH than for PDMS M by almost 2°C. A big difference in melting enthalpy of those samples (nearly 80%) was also observed. The lowest value is noticed for PDMS HPH sample, the biggest for PDMS M. The variation is also significant in C_p_ value for PDMS K sample. The C_p_ values obtained for PDMS M and PDMS HPH agreed with the reference sample C_p_. No serious changes are present for T_g_ and ΔC_p_ values. Nevertheless, the change of thermograms’ shape indicates strong impact of the production technology. Therefore, it can be assumed that the dispersion degree resulting from the production technology significantly affects the polymer chain mobility. The higher T_m_ for PDMS HPH can be indicative of less defective crystallites, which might have grown during cold crystallization. This process can take place if during the initial cooling the polymer crystallization was hindered, in this case because of polymer-filler interaction^22^. Another possible explanation of that phenomenon is that the production technology could have impact on the cross-linking level of the nanocomposite, which again influences crystallization dynamics^23^.

Figures 4b and S3 (see: Supporting information) show TGA thermograms of samples made by three previously described routes as well as reference TGA thermograms of neat PDMS and graphene used as a filler. The neat PDMS starts to decompose in approximately 360°C, the second decomposition step can be observed in about 496°C and third in 537°C. Table 1 presents temperatures of maximum degradation rates (denoted as T_max1_–T_max4_) obtained from thermograms from Fig. 4b. According to those values, clear enhancement in thermal stability is seen since in nanocomposites each step of decomposition begins in higher temperatures than in the neat PDMS. What is more, four nanocomposite decomposition steps are noticed unlike in neat PDMS. Depending on production technology, the first decomposition step begins in the range of ca. 393–450°C. The decrease of that temperature correlates with the rise of sample homogeneity revealed by Raman mapping and microscopic techniques. The second and third decomposition steps start around 500°C and within 550–580°C range respectively. No regularity of temperature variation is visible here. Fourth decomposition step is observed within 836–839°C range and the variation is insignificant.

Generally, neat PDMS chains take the form of spirals, which in higher temperatures tend to warp and close, creating cyclic compounds. However, interaction with a filler can increase the thermal stability of the polymer, changing the chain-ring equilibrium^24,25^. The interfacial polymer fraction is known to have modified structure, slower dynamics and enhanced thermal stability^26^. Since presence of well-dispersed nanoparticles increases its amount in the material, adding graphene to PDMS significantly affects decomposition temperature of the whole nanocomposite. Another factor that could affect thermal stability is increased thermal conductivity of the more homogeneous nanocomposite. The facilitated heat flow could lead to more efficient heat dissipation within the material. Such an effect was observed by Kong et al.^27^, but in their case a single manufacturing method was used. It must be noticed that different processing techniques can damage the filler’s crystalline structure in different ways and better dispersion does not necessarily translate to higher overall thermal conductivity.

Based on the results presented above, the microfluidization method turns out to be the most promising route in terms of achieving the highest filler dispersion homogeneity level. Therefore, next, structural and thermal properties investigations of PDMS-graphene nanocomposites prepared this way as a function of filler concentration was exectuted.

**Figure 5:**
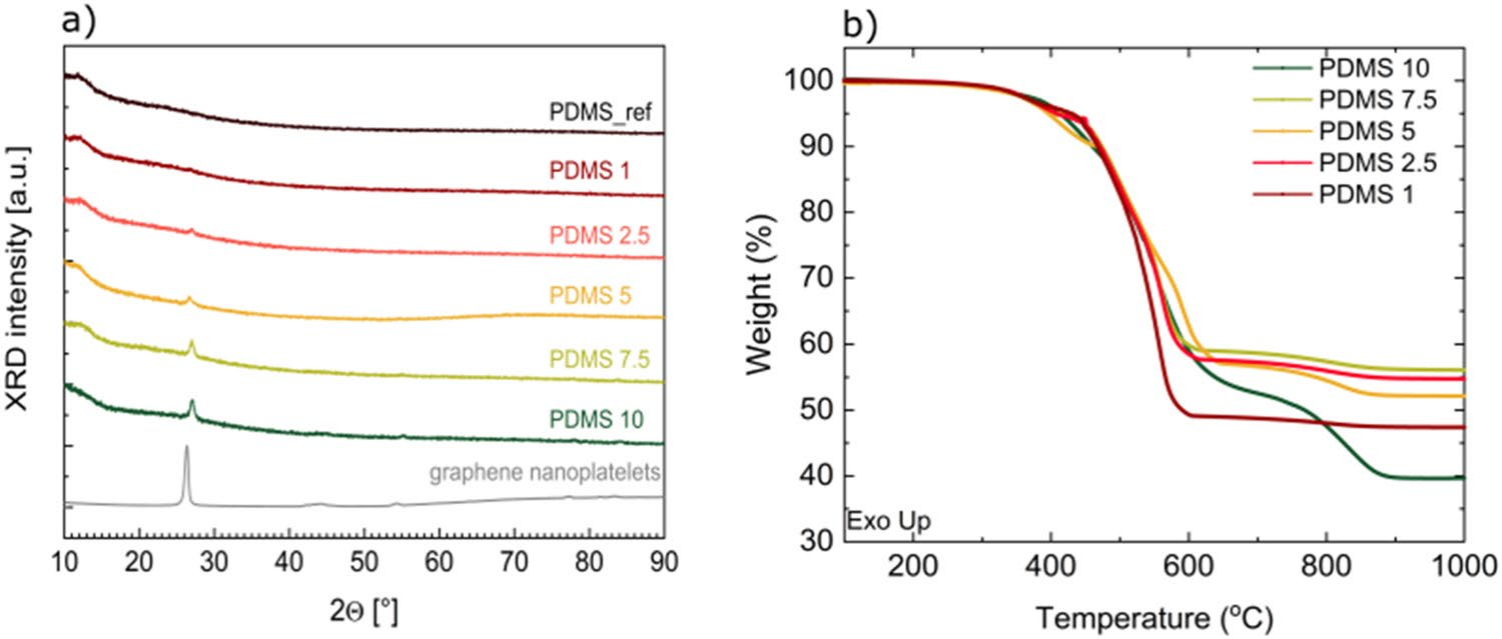
(**a**) Diffractograms of pure PDMS (grey curve) and nanocomposites with different graphene addition (green to blue). The pattern of the graphene nanoplatelets (black curve) was given for comparison. (**b**) Typical TGA thermogram of a graphene/PDMS nanocomposite. Numbers (1–10) stands for respective wt% graphene loading.

X-ray diffractometry was used to verify the amorphousness of the samples. The results are presented in Fig. 5a. In the diffractogram of pure PDMS (black curve) no peaks are observed, indicating that the sample was amorphous. In samples with the graphene filler, low-intensity peaks at 2θ = 27°, 55° and 83.9° emerged and their intensity increased with filler content (red to green curves). These positions were ascribed to graphene filler (grey curve), which allowed us to conclude that these peaks do not come from any structural changes in the nanocomposite.

To reveal changes in thermal properties induced by filler concentration variation, the TGA and DSC analyses were performed. Figure 5b and Table 2 present TGA thermograms, final weights, and temperatures of maximum degradation rate for PDMS samples of different graphene concentration. The samples of lower graphene concentration decompose to smaller extent than neat PDMS, their final weights fluctuate around 50 to almost 60%. The exception is PDMS 10 sample, whose weight after decomposition is comparable to neat PDMS. When it comes to temperatures of maximum degradation rate, excluding T_max2_ they shift up significantly in comparison with the neat polymer and in majority of cases the highest T_max_ temperatures are observed for samples with the highest graphene concentration. However, there is no obvious trend that could describe T_max_- filler concentration relationship. This can be a result of still imperfect material homogenization. Although microfluidization can be considered a promising manufacturing method, nanocomposites produced this way are not completely free from agglomeration of the nanofiller and microstructure irregularities. Another possible explanation is that there are other factors affecting the polymer structure rearrangements and stability. However, this issue requires deeper analysis.

**Table 2:** Summary of thermogravimetry measurements of graphene/PDMS nanocomposites of 1–10%wt graphene concentration.

Sample	Final weight [%]	T_max1_ [°C]	T_max2_ [°C]	T_max3_ [°C]	T_max4_ [°C]
PDMS neat	41.7	359.87	495.88	536.49	–
Graphene	0	400.83	718.22	–	–
PDMS 1	51.0	364.19	495.33	558.30	805.28
PDMS 2.5	58.8	380.00	479.34	563.42	808.58
PDMS 5	58.6	407.70	507.73	590.61	820.95
PDMS 7.5	58.1	382.53	489.29	559.87	805.98
PDMS 10	39.6	450.85	505.16	553.75	836.55

*T*_*max1-4*_ maximum degradation rate temperatures.

**Figure 6:**
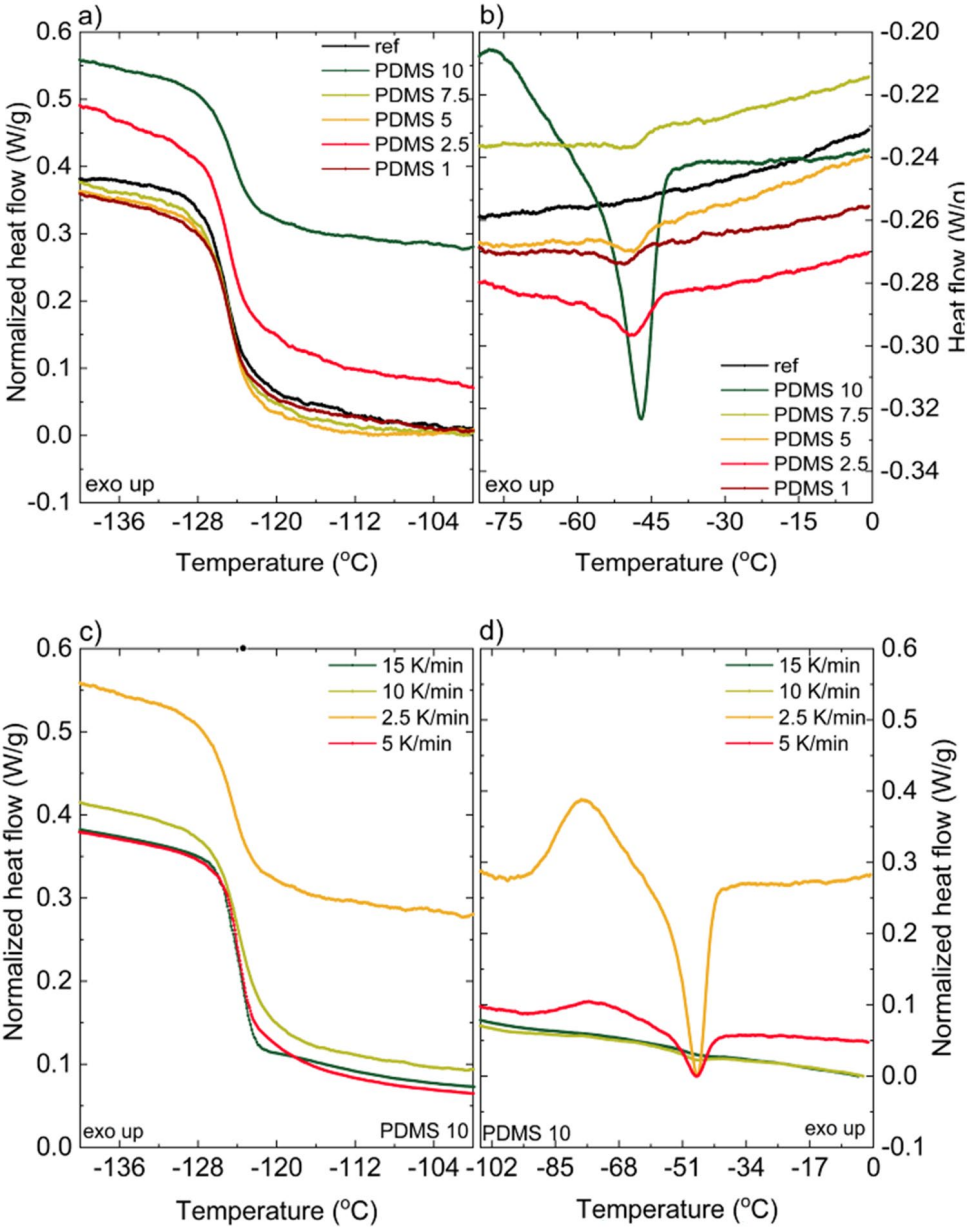
(**a**,**b**) DSC thermograms of graphene/PDMS nanocomposite of 1–10wt% graphene concentration at the constant heating rate of 2.5°C/min.: the glass transition region (**a**), melting peaks (**b**), (**c**,**d**) DSC thermograms of 10%wt graphene/PDMS nanocomposite at different heating rates showing glass transition region (**c**) and melting peaks (**d**).

Figure 6 shows the sections of thermograms showing (a) glass transition region and (b) melting peaks obtained for heating rate 2.5°C/min. Table 3 summarizes the results based on the thermograms for that heating rate. The change of glass transition temperature is negligible (less than 1%). Specific heat capacity (ΔC_p_) changes approximately by 20% (comparing the highest value obtained for the neat polymer and for the nanocomposite of 5%wt graphene concentration). An important notice is that the neat PDMS was prepared according to the producers’ instruction, not via microfluidization. Next, it is observed that the increase in melting temperature reaches approximately 3°C. Such behavior could have origin in spatial confinement of macroparticles induced by interaction with the nanofiller^28^. The increase of crystallization temperature by over 10°C is also observed and several explanations of this phenomenon could be distinguished. One of them is again the effect of spatial confinement of the matrix imposed by nanofiller particles and consequent limitation in the chain mobility^28^. Another reason may be the presence of intrinsic stress induced by the nanofiller and resulting increased surface tension of the grain boundaries^29^. The crystallization temperature could increase so significantly also because the presence of the filler may limit the crystallization nucleation as the crystallization does not occur close to the filler surface^22^. To the best of our knowledge the only known filler that can facilitate PDMS crystallization is carbon nanotubes^23^.

**Table 3:** Summary of typical values coming from DSC measurement of graphene/PDMS nanocomposites of 1–10 wt% graphene concentration.

k = 2.5 °C/min	T_g_ [°C]	ΔC_p_ [J/(g °C)]	T_m_ [°C]	H_m_ [J/g]	T_c_ [°C]	H_c_ [J/g]	C_p_ [J/(g °C)]
PDMS neat	− 125.18	0.3804	–	–	–	–	1.4600
PDMS 1	− 124.87	0.3284	− 50.51	0.2724	− 88.36	0.1325	1.4129
PDMS 2.5	− 124.87	0.3349	− 48.92	0.9704	− 87.29	0.5500	1.5082
PDMS 5	− 124.66	0.3040	− 48.48	0.3740	− 79.86	0.1952	1.3776
PDMS 7.5	− 124.71	0.3380	− 48.58	0.2116	− 80.90	0.3167	1.4176
PDMS 10	− 124.45	0.3159	− 47.11	3.1635	− 77.68	4.2918	1.4600
2.5 K/min	− 124.45	0.3159	− 47.11	3.1635	− 77.68	4.2918	–
5 K/min	− 123.87	0.3213	− 47.26	0.8169	− 75.86	0.4847	–
10 K/min	− 124.00	0.3555	− 47.02	0.1360	–	–	–
15 K/min	− 123.34	0.3731	− 46.61	0.1227	–	–	–

The heating rate remained constant: 2.5 °C/min. C_p_ was measured in quasi-isothermal mode. The second part of the table shows a summary of typical values coming from DSC measurement for samples with constant 10%wt graphene concentration. The heating rate changed from 2.5 to 15 K/min.

*T*_*g*_ glass transition, *ΔC*_*p*_ heat capacity change, *T*_*m*_ melting temperature, *H*_*m*_ melting enthalpy, *C*_*p*_ heat capacity.

Interestingly, a sharp change in the shape of PDMS 10 thermogram is observed (Fig. 6b), which indicates higher crystallization degree. As the 10 wt% graphene concentration is high (regarding to volume of the filler), the whole mixture after the solvent evaporating (see: Experimental section, description of sample production) became sticky and dense. Despite the effort put in the sample preparation, the imperfection of curing agent incorporation in such dense mixture cannot be excluded. If such a situation had occurred, the cross-linking level of the sample is lowered compared to other samples and it would explain the change of thermogram in this specific case.

Figure 6c,d and Table 3 show the results obtained for the PDMS 10 sample with different heating rates: 2.5–15 K/min. The glass transition temperature value increases with the heating rate, which is typical for all polymers^30,31^. Similarly, the small increase of ΔC_p_ and the decrease of melting enthalpy is considered typical for all polymers. A slight increase of melting temperature is also observed. Classically, the melting temperature should remain insensitive to the changing heating rate^29,32^. The observed change is rather unintuitive—one could assume that higher heating rate could result in quicker crystallite nucleation and growth (during cold crystallization), which should lead to smaller, more defective crystallites, melting in lower temperatures. The opposite observation clearly needs further investigation.

## Conclusions

Within this work, PDMS/graphene nanocomposites were prepared using three different routes: simple magnetic mixer stirring, calendaring and microfluidization (high pressure homogenization), while keeping the graphene concentration constant (10 wt%). Based on imaging techniques, Raman mapping, DSC and TGA, it has been proven that the selection of production technology has a great impact on the filler distribution in the matrix and the properties of the final nanocomposite. The most homogeneous filler distribution resulting in the most significant filler-matrix interactions was obtained by microfluidization, which may be associated with applying high shear stress to the mixture.

In the second part of the study the microfluidization method was used to prepare nanocomposites of different graphene content. The filler concentration influence had been investigated in terms of structural and thermal properties. It has been shown that the concentration variation affects the PDMS chain mobility, cross-linking level, grain sizes and/or number of defects in the nanocomposite.

The results confirm that addition of graphene nanoplatelets can be used to enhance thermal stability of PDMS. Its efficiency depends on filler-matrix interactions, which can be intensified by improving the filler dispersion (via different processing methods) and/or increasing filler content. However, the latter additionally has a strong impact on other properties of the material (e.g. viscosity of the filler-resin mixture), which limits the range of concentrations which can be considered.

## Methods

### Samples

Polydimethylsiloxane (PDMS) under the trade name Sylgard 184 (Dow Corning, USA) was employed as a nanocomposite matrix. The filler used was graphene nanoplatelets called as xGnP H-5 (XG Sciences, Inc., USA, 5 µm particle size). In some of the processes isopropanol (Carl Roth, ≥ 99.8%) was utilized.

In the first part of the study samples containing 10 wt% graphene nanoplatelets were made using three approaches. The magnetic mixer stirring (sample PDMS M) involved the polymer and graphene blending for 5 h using a magnetic stirrer with hotplate heated to 45°C and subsequent, manual mixing with an appropriate amount of curing agent. The mixture was poured into Petri dishes, degassed under reduced pressure for 1 h and cured at 100°C for another 1 h. Sample PDMS K was prepared in a similar manner except before adding the curing agent the mixture was calendared using Exact 50 I (3 passes). As calendaring was chosen as a comparative method aiming mainly at showing the filler dispersion changes as a general for this specific method and a simple model of the equipment with limited control of the roller spacing was used, the calculation of applied forces was neglected. Sample PDMS HPH was prepared via microfluidization. The mixture of PDMS and graphene nanoplatelets was diluted with isopropanol to decrease its viscosity and passed through GEA Lab Homogenizer PandaPLUS 2000 (see Fig. S4 for detailed scheme) 15 times under pressure rising from 100 to 800 bar. Then the solvent was fully evaporated on a magnetic stirrer with a hotplate heated to 80°C, the mixture was poured into Petri dish and cured at 100°C for 1 h. In all approaches, publications^33–36^ in these fields do not require detailed computational approach to the mechanisms taking place in devices, all the more so it may depend on factors such as viscosity or particle size and thickness. Thus, the applied forces calculation was skipped also to this method.

In the second part of the study samples of various graphene concentrations (1, 2.5, 5, 7.5, 10 wt%) were produced using microfluidization. The procedure was the same as described above for 10%wt graphene concentration.

### Methods

The Raman measurements were performed using Renishaw inVia Raman spectrometer. The laser wavelength of λ = 532 nm, the diffraction grading of 1800 l/mm in backscattering geometry were used. The Raman maps were run collecting 1560 spectra in area 51 × 31 µm with step 1.5 µm for each sample. All measurements were carried out at room temperature, keeping the laser power at a sample-safe level. Each spectrum within a map was fitted by Lorentzian function to obtain modes’ parameters, especially position and intensity.

Thermal properties of nanocomposites were studied by differential scanning calorimetry technique (DSC), using TA Instruments Q2000 calorimeter with liquid nitrogen cooling system. To measure glass transition (T_g_), crystallization (T_c_) and melting (T_m_) temperatures and enthalpies as well as heat capacity change at T_g_, the samples, initially kept at 25 °C, were equilibrated at − 160 °C and then heated to 0 °C with different heating rates (2.5, 5, 10, 15°C/min). The modulated temperature DSC (MT-DSC) measurements of heat capacity were performed at 20 °C in a quasi-isothermal mode with an amplitude of temperature modulation equal to 1 °C and modulation period of 60 s. The measurements were taken in high purity (99.999%) helium flow (25 ml/min). Samples of investigated materials were carefully weighed with an accuracy of 0.01 mg and loaded into standard aluminum pans. The typical mass of the sample was about 5 mg. Before the measurements, instrumental baseline and heat flow were calibrated according to a standard procedure involving a reference sapphire sample. The cell constant and temperature calibration were performed using pure (99.999%) indium.

Thermogravimetric analysis (TGA) was performed using TA Instruments SDT Q600. Samples weighing an average of 10 mg (8–16 mg) were heated in air from room temperature to 1100°C at a constant rate of 20°C/min. The measurements were taken in air flow of 100.0 ml/min.

Structural investigations were performed using X-ray diffractometry (XRD) on Malvern Panalytical diffractometer working in Bragg–Brentano geometry in reflection mode. The samples were irradiated with Cu Kα wavelength (λ = 0.15406 nm). To ensure the homogeneity, samples were rotated during measurement with the revolution period equal to 8 s. Data was collected in room temperature, in 2θ = 10–90° range. The peak position calibration was performed before measurement using silicon standard.

## Supplementary Information

Supplementary Information.

## Data Availability

The datasets used and/or analyzed during the current study are available from the corresponding author on reasonable request.
