# Supplementary material for: Influence of the filler distribution on PDMS-graphene based nanocomposites selected properties
Source: Sci Rep. 2022 Nov 9;12:19038. doi: 10.1038/s41598-022-23735-3 (PMC9646694; doi:10.1038/s41598-022-23735-3)
Supplement: Supplementary file 1 — Supplementary Information. [file 41598_2022_23735_MOESM1_ESM.docx]

**SUPPORTING INFORMATION**

**Influence of the Filler Distribution on PDMS-Graphene Based Nanocomposites Selected Properties**

**Anna Łapińska^1^, Natalia Grochowska^2^, Jerzy Antonowicz^1^, Przemysław Michalski^1^, Kamil Dydek^2^, Anna Dużyńska^1^, Agata Daniszewska^1^, Milena Ojrzyńska^1^, Klaudia Zeranska^1^, Mariusz Zdrojek^1^**

^1^ Warsaw University of Technology, Faculty of Physics, Koszykowa 75, 00-662 Warsaw, Poland

^2^Warsaw University of Technology, Faculty of Material Science and Engineering, Wołoska 141, 02-507 Warsaw, Poland


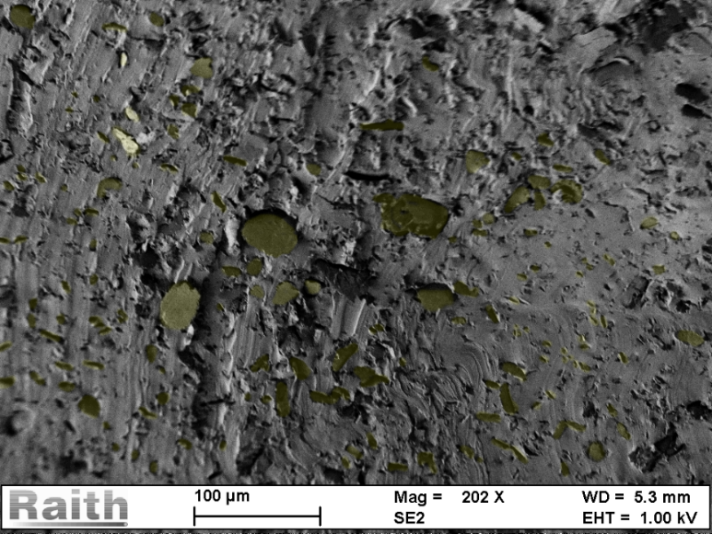


*Fig.S1: SEM scan of graphene/PDMS composite produced by mechanical mixing, PDMS M.*


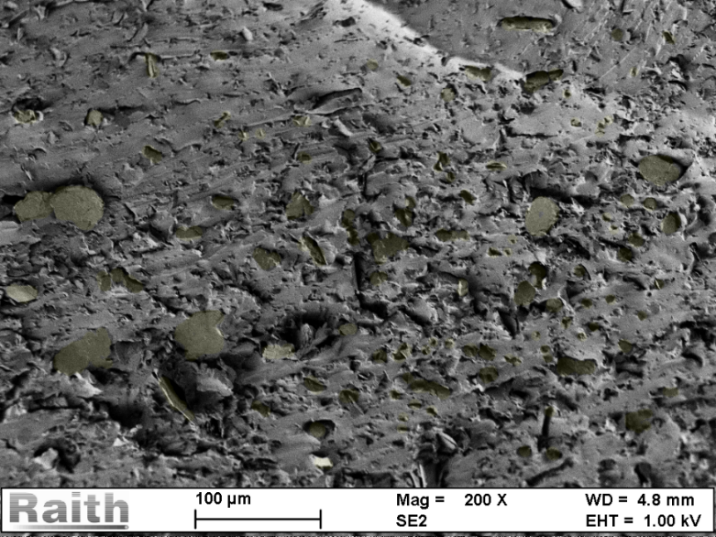


*Fig.S2: SEM scan of graphene/PDMS composite produced by calendaring, PDMS K.*


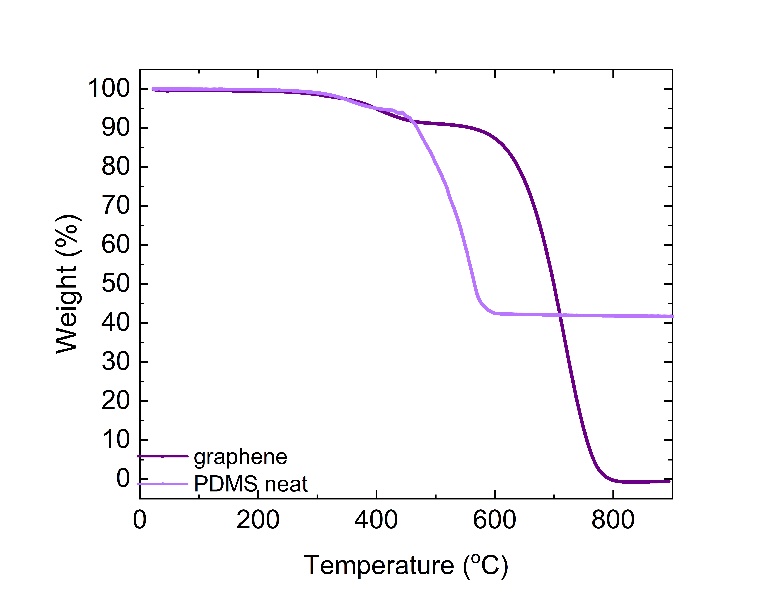


*Fig. S3: TGA curves obtained for graphene and PDMS reference samples.*

*
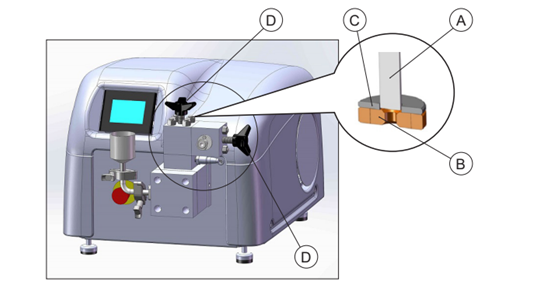
*

*Fig. S4: Fig. 1. GEA Lab Homogenizer PandaPLUS 2000 [source: GEA Instructions Manual]*

Homogenization of polymer and graphene flakes in high pressure homogenizers involves such phenomena as mixing, shearing and cavitation. The homogenizer used in this experiment is GEA Lab Homogenizer PandaPLUS 2000 with a homogenization module presented in Fig. S4 with the most common homogenizing elements setup used in nanomaterials processing. The homogenization module has three main elements – A) an impact head, B) a passage head, C) an impact ring. The setup includes a cooling system. Parameters of the process, which can be controlled and have an impact on the final form of product are a number of passes through the system (or time) and the value of input pressure. The bigger the magnitude of input pressure - the smaller the distance between the passage head, the impact ring and the impact head.

*Table S1: Summary of values obtained from DSC measurements for heating speeds: 5, 10 and 15 K/min.*

| k = 5 K/min | Tg [˚C] | ΔCp [J/(g·°C)] | Tm [˚C] | Hm [J/g] | Tc [˚C] | Hc [J/g] |
| --- | --- | --- | --- | --- | --- | --- |
| PDMS neat | -124.49 | 0.3735 |  |  | - | - |
| PDMS 1 | -124.29 | 0.3620 | -52.01 | 0.0543 | -76.70 | 0.0238 |
| PDMS 2.5 | -124.13 | 0.3481 | -47.90 | 0.1990 | -81.22 | 0.1866 |
| PDMS 5 | -124.03 | 0.3361 | -46.94 | 0.0350 | -82.56 | 0.0741 |
| PDMS 7.5 | -124.16 | 0.3442 | -49.20 | 0.0384 |  |  |
| PDMS 10 | -123.87 | 0.3213 | -47.26 | 0.6944 | -75.86 | 0.4120 |

| k = 10 K/min | Tg [˚C] | ΔCp (step) [J/(g·°C)] | Tm [˚C] | Hm [J/g] | Tc [˚C] | Hc [J/g] |
| --- | --- | --- | --- | --- | --- | --- |
| PDMS neat | -124.51 | 0.3546 |  |  | - | - |
| PDMS 1 | -124.49 | 0.3768 |  |  |  |  |
| PDMS 2.5 | -123.78 | 0.3708 | -47.44 | 0.09619 |  |  |
| PDMS 5 | -124.49 | 0.3434 |  |  |  |  |
| PDMS 7.5 | -123.07 | 0.3765 |  |  |  |  |
| PDMS 10 | -124.00 | 0.3555 | -47.02 | 0.1156 |  |  |

| k = 15 K/min | Tg [˚C] | ΔCp (step) [J/(g·°C)] | Tm [˚C] | Hm [J/g] | Tc [˚C] | Hc [J/g] |
| --- | --- | --- | --- | --- | --- | --- |
| PDMS neat | -124.10 | 0.3501 |  |  | - | - |
| PDMS 1 | -123.93 | 0.3744 |  |  |  |  |
| PDMS 2.5 | -124.19 | 0.3738 |  |  |  |  |
| PDMS 5 | -124.45 | 0.3576 |  |  |  |  |
| PDMS 7.5 | -123.91 | 0.3795 |  |  |  |  |
| PDMS 10 | -123.34 | 0.3731 | -46.61 | 0.1043 |  |  |
